# Perceived Stress and Associations Between Food Insecurity, Diet Quality, and Eating Behaviors: Evidence from Two Cross-Sectional Studies in U.S. Samples

**DOI:** 10.3390/nu18071153

**Published:** 2026-04-03

**Authors:** David G. Figueroa, Athena Cisneroz, Caroline A. Stiver, Lauren Tiongco-Hofschneider, Barbara A. Laraia, Elissa S. Epel, A. Janet Tomyiama

**Affiliations:** 1 Department of Psychology, University of California, Los Angeles, Los Angeles, CA 90095, USA; 2 Psychology and Human Services Department, Los Angeles City College, Los Angeles, CA 90029, USA; hofschlt@lacitycollege.edu; 3 Division of Community Health Sciences, University of California, Berkeley, Berkeley, CA 94720, USA; 4 Department of Psychiatry and Behavioral Sciences, University of California, San Francisco, San Francisco, CA 94107, USA

**Keywords:** food insecurity, psychological stress, eating behaviors, diet quality

## Abstract

**Background/Objectives:** The present investigation examined whether perceived stress statistically mediated the association between food insecurity and diet quality, as well as maladaptive eating behaviors (i.e., reward-based eating, comfort eating). **Methods:** Study 1 used cross-sectional data from the National Heart, Lung, and Blood Institute Growth and Health Study (*N* = 624), in which Black and white women completed self-report measures of food security, perceived stress, diet quality, and reward-based eating. Study 2 used cross-sectional data from a census-matched U.S. sample by age, gender, income, race/ethnicity, and census region (*N* = 1993), with self-report measures of food security, perceived stress, and comfort eating. Mediation analyses tested the indirect effect of perceived stress on associations between food insecurity and diet quality, reward-based eating, and comfort eating, controlling for sociodemographic factors. **Results:** In Study 1, food insecurity was positively correlated with perceived stress (*r* = 0.30) and negatively correlated with diet quality (*r* = −0.11). Perceived stress mediated the relationship between food insecurity and higher reward-based eating (indirect effect = 0.14, 95% CI [0.08, 0.22]) but did not mediate the association between food insecurity and diet quality (indirect effect = −0.04, 95% CI [−0.11, 0.03]). In Study 2, food insecurity was positively correlated with perceived stress (*r* = 0.42) and comfort eating (*r* = 0.19). Using a two-part mediation model, perceived stress mediated the association between food insecurity and the frequency of comfort eating among individuals who reported at least one day of comfort eating, with the strongest indirect effect observed among food-insecure individuals (conditional indirect effect = 0.75, 95% CI [0.49, 1.13]). **Conclusions:** Across two cross-sectional studies, higher perceived stress statistically mediated the relationship between food insecurity and two forms of maladaptive eating behaviors, suggesting that perceived stress is an important correlate of these relationships. Future work is needed to further evaluate the causal relationships between these constructs.

## 1. Introduction

Nearly 18.3 million households in the U.S. experienced food insecurity in 2024, a condition that is defined by the United States Department of Agriculture as a “household-level economic and social condition of limited or uncertain access to adequate food” [1,2]. Food insecurity is not only a nutritional limitation but may also be a psychosocial stressor, with consequences that extend beyond inadequate food access and intake. Seligman and Schillinger argue that food insecurity creates unique psychosocial risks beyond material deprivation, some of which may operate through stress [3]. Among potential stress pathways is perceived stress or the subjective appraisal that environmental demands are threatening and exceed coping resources [4]. Perceived stress may be especially relevant for diet quality and eating behaviors [5]. Consistent with this view, studies across diverse populations, including college students [6], younger adults [7], older adults [8], caregivers [9], pregnant women [10], and lower-income families [11,12], have reported a positive relationship between food insecurity and perceived stress.

**Figure 1:**
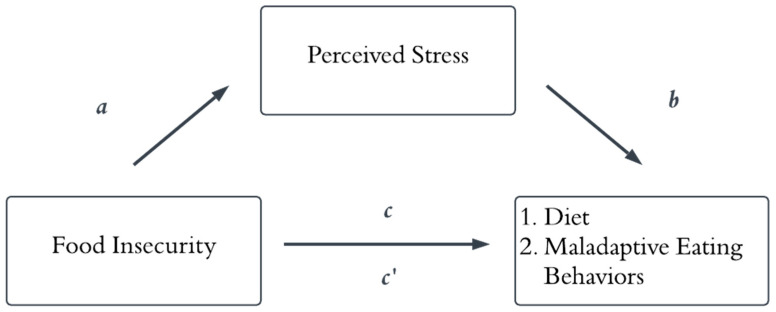
Conceptual mediation model linking food insecurity to diet- and eating-related outcomes via perceived stress. Note: The conceptual model depicts perceived stress as a statistical mediator of the association between food insecurity and diet- and eating-related outcomes. Specifically, higher levels of food insecurity are associated with greater perceived stress (*a* path), which in turn is associated with poorer diet quality and maladaptive eating behaviors (*b* path). Although not the primary focus of the current analyses, the model also includes the total (*c*) and direct (*c′*) associations between food insecurity and diet- and eating-related outcomes.

Perceived stress has been examined as a statistical mediator linking food insecurity to diet and eating behaviors. Among a sample of women, perceived stress explained the association between food insecurity, eating disorder pathology, and binge eating-related traits [13]. In a sample of Latinx participants, greater perceived stress mediated the association between food insecurity and higher levels of emotional eating [14]. Perceived stress has also been implicated in dietary outcomes, with one study showing that it mediated the relationship between food insecurity and a higher consumption of sugar-sweetened beverages among young adults [15]. These studies provide preliminary evidence that perceived stress may serve as a statistical mediator between food insecurity and diet- and eating-related outcomes (Figure 1). In the current examination of this framework, two different forms of eating behavior are tested. Reward-based eating refers to patterns of eating driven by heightened responsiveness to palatable foods, including a lack of control, reduced satiety, and preoccupation with food, and may occur in the absence of negative affect [16]. In contrast, comfort eating refers specifically to eating in response to negative emotions as a coping strategy [17]. Although these constructs are related and may co-occur, they reflect distinct behavioral responses and may differentially relate to stress-related mechanisms. The current cross-sectional work extends prior research by examining this conceptual framework across distinct outcomes and samples.

### Overview of Study Aims

Across two studies, we tested a shared conceptual framework in which perceived stress statistically mediated associations between food insecurity and diet- and eating-related outcomes. The studies were not intended as direct replications. Instead, they were designed as complementary tests of the conceptual model across samples and across related, but distinct, outcomes, including diet quality, reward-based eating, and comfort eating. Study 1 relied on a sample of Black and white women from the National Heart, Lung, and Blood Institute Growth and Health study (NGHS) to test the hypothesis that stress would mediate the association between food insecurity and diet quality and reward-based eating. Study 2 relied on a census-matched U.S. sample from the Eating in America Study to test the hypothesis that perceived stress would mediate the association between food insecurity and more frequent comfort eating among a U.S. census-matched sample. The indirect effect of perceived stress was hypothesized to remain significant even after controlling for key sociodemographic variables like income, education, and household size.

## 2. Study 1

The first aim of Study 1 was to investigate whether greater perceived stress statistically mediated the association between food security status and poor diet quality. Poor diet quality is characterized by a lower intake of vegetables and whole grains and greater consumption of foods and drinks with added sugars [18]. Some existing research shows that food insecurity is directly associated with poor diet quality [19]. For instance, adults living below 300% of the federal poverty line who were food-insecure reported a higher consumption of high-fat dairy products, salty snacks, and sugar-sweetened beverages [20], with these associations being particularly pronounced among non-Hispanic White and Asian individuals [21]. Similarly, emerging adults who were food-insecure consumed fewer vegetables and whole grains and more foods and drinks high in added sugars [18]. A systematic review reported an inverse relationship with food insecurity and diet quality, where adults who reported food insecurity consumed less vegetables, fruits, dairy, vitamins, and minerals compared to those who reported no food insecurity [22]. Therefore, Study 1 tested whether perceived stress statistically mediated the association between food insecurity and diet quality.

The second aim of Study 1 was to examine whether perceived stress statistically mediated the association between food insecurity and a form of maladaptive eating, reward-based eating. Reward-based eating is characterized by a lack of control over eating, reduced satiety from eating, and preoccupation with food. In human and non-human animal models, the consumption of foods rich in fat, sugar, and salt is highly rewarding, engaging areas in the brain like the amygdala, insula, and nucleus accumbens [23]. Stress could elicit reward-based eating by triggering the reward networks associated with finding pleasure in overeating [16,17,24]. One study showed that rats assigned to a food-insecure eating schedule consumed more palatable liquid and displayed increased motivation to earn sucrose when fed a high-fat/high-sugar diet, compared to rats assigned to a food-secure eating schedule [25]. In the context of food insecurity among humans, individuals may seek out highly palatable, rewarding foods such as ultra-processed foods [26] and soft drinks [27], which have the potential to induce chronic disease risk [28,29]. To our knowledge, research has yet to examine the associations between food insecurity, perceived stress, and reward-based eating in humans. The current study sought to address this gap in the literature by testing perceived stress as a potential statistical mediator.

### 2.1. Study 1 Materials and Methods

#### 2.1.1. Analytic Sample

Study 1 analyzed data from NGHS, a longitudinal prospective cohort study originally designed to examine the development of cardiovascular risk factors and obesity in Black and white girls [30]. Eligibility criteria for the first-wave cohort are discussed in detail elsewhere [30]. In summary, participants were recruited from areas identified from census tract data as having an equal proportion of Black and white children with the least socioeconomic disparity between the two racial groups. Study 1 utilized data from the 2016 follow-up assessment of the Richmond, California, cohort when food insecurity data was collected. Eligible participants were (1) enrolled in the original NGHS; (2) not pregnant when recruited to participate in the follow-up assessment and did not experience a pregnancy, miscarriage, or abortion three months prior to recruitment; and (3) not incarcerated or living abroad at the time of this study. Six hundred and twenty-four women (*n* = 307 Black and *n* = 317 white) were enrolled in the 30-year follow-up assessment (*M_age_* = 39.52; *SD* = 1.43). Table 1 displays sample characteristics distinguished by food security status.

#### 2.1.2. Measures

Food Insecurity

Food insecurity was measured using the USDA 18-item U.S. Household Food Security Survey Module [31]. The survey measured disruptions in eating patterns in the past year for households with and without children (e.g., “We worried whether our food would run out before we got money to buy more”). Categories ranged from high food security (0) to very low food security (i.e., 6–10 for households without children; 8–18 for households with children). Food insecurity was dichotomized in the current study, where a score of zero reflected food security, and a score of one or more reflected food insecurity. This decision was based on the literature showing that even marginal levels of food insecurity are correlated with poor health outcomes relative to that of those who are food-secure [32].

Perceived Stress

Participants completed the 10-item version of the Perceived Stress Scale [33]. This measure assesses personal feelings of stress across various situations in the past month. An example item is “How often have you felt that you were unable to control the important things in your life?” Items were anchored on a 0 (“Never”) to 4 (“Very Often”) scale, with higher scores indicating greater perceived stress (α = .78).

Diet Quality

Participants completed a 3-day record of their diet, and an Alternate Healthy Eating Index-2010 (AHEI-2010) score was derived from these records. The AHEI-2010 evaluates how closely an individual adheres to dietary guidelines associated with lower disease risk. Scores range from 1 to 110, with higher scores indicating better adherence to dietary guidelines [34]. The final score reflects AHEI-2010 scores that were averaged across the 3 days of food records.

Reward-Based Eating

The 9-item Reward-Based Eating Drive scale [16] assessed the strength of reward-based motivations for eating, including a lack of control over eating, preoccupation with food, and lack of satiation (e.g., “When it comes to foods I love, I have no will power.”). Items were presented on a 0 (“Strongly disagree”) to 4 (“Strongly agree”) scale. Higher average scores reflected a greater reward-based eating drive (α = .80).

#### 2.1.3. Covariates

Several variables were included as covariates to address potential confounding influence on focal variables. To rule out potential socioeconomic confounds, income and education from the 30-year follow-up assessment were added as covariates [35]. Furthermore, older age [36,37,38], belonging to a racial/ethnic minority group [39,40,41], and household size [42] are associated with an increased risk of food insecurity. Therefore, age, race/ethnicity, and household size were included as covariates. All covariates were assessed when respondents were in adulthood.

#### 2.1.4. Statistical Analyses

Hypotheses and data analytic plans were pre-registered (OSF; https://osf.io/uwr92/overview, accessed on 21 October 2021). Zero-order correlations were conducted to examine the relationship between all variables of interest. Missing data were addressed using Bayesian multiple imputation implemented in rBlimp (0.2.25). Mediation models were also estimated in rBlimp following a standard mediation framework. Food insecurity status was included as a binary predictor and perceived stress as a continuous mediator. Diet quality and reward-based eating were included as outcomes in separate models. In these models, the indirect effect represented the product of the association between food insecurity and perceived stress (path *a*) and the association between perceived stress and the outcome (path *b*). In the context of this study, the indirect effect reflects the extent to which the association between food insecurity and the outcome is statistically explained by perceived stress. Models included age, race, household size, income, and education as covariates. Race and education were dummy-coded. The models were estimated using 20 imputations to ensure stable parameter estimates under missing data, with 5000 burn-in cycles followed by 10,000 posterior iterations across two Markov Chain Monte Carlo chains. Indirect, direct, and total effects were estimated. An indirect effect was considered statistically significant if the 95% credible interval did not contain a null value of zero. Whereas frequentist confidence intervals reflect the proportions of intervals that would contain the population parameter across repeated samples, the Bayesian credible interval represents the probability that the parameter lies within the interval given the observed data. Both confidence intervals and credible intervals similarly assess whether parameter estimates differ from zero. The magnitude of the indirect effect was quantified using the squared standardized indirect effect [43], which reflects the proportion of variance in the outcome explained by the mediator. At the recommendation of a reviewer, sensitivity analyses were conducted using the full food insecurity scale specified as an ordinal predictor rather than a dichotomized predictor. The results were substantively consistent, with no meaningful changes in statistical significance or interpretation. Accordingly, the results from models using the dichotomized food insecurity variable are presented.

### 2.2. Study 1 Results

**Figure 2:**
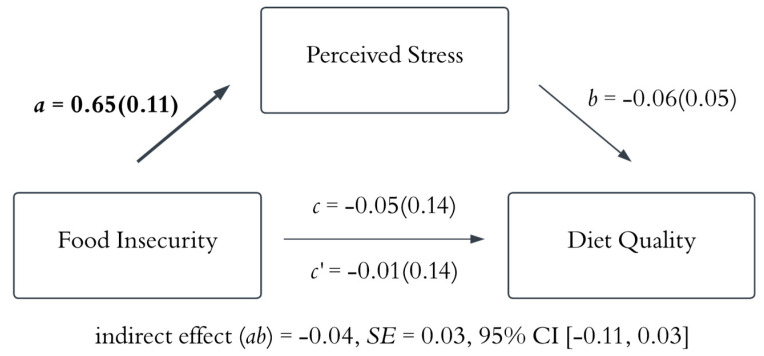
The mediation model of the relationship between food insecurity and diet quality. Note: The results indicated that food insecurity was positively associated with perceived stress, but perceived stress was not significantly associated with diet quality. The indirect effect of perceived stress was not significant. The *a* path represents the association between food insecurity and perceived stress. The *b* path represents the association between perceived stress and diet quality. The *c* path represents the total effect of food insecurity on diet quality without controlling for the mediator. The *c’* path represents the direct effect of food insecurity on diet quality. The values reported are standardized coefficients with standard errors in parentheses. Bold indicates statistical significance (the 95% credible interval does not contain zero). SE = standard error; CI = credible interval.

**Figure 3:**
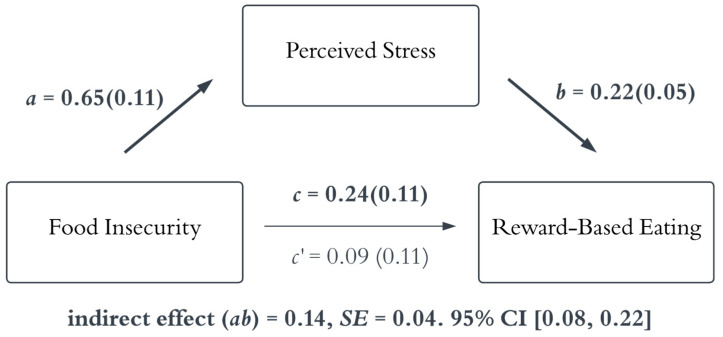
The mediation model of the relationship between food insecurity and reward-based eating. Note: The results indicated that food insecurity was positively associated with perceived stress, and perceived stress was significantly associated with higher reward-based eating. The indirect effect of perceived stress was significant. The magnitude of the indirect effect was small (υ = 0.02) per Cohen’s [44]) benchmarks. The *a* path represents the association between food insecurity and perceived stress. The *b* path represents the association between perceived stress and reward-based eating. The *c* path represents the total effect of food insecurity on reward-based eating without controlling for the mediator. The *c’* path represents the direct effect of food insecurity on reward-based eating. The values reported are standardized coefficients with standard errors in parentheses. Bold indicates statistical significance (the 95% credible interval does not contain zero). SE = standard error; CI = credible interval.

Table 2 displays zero-order correlations. Food insecurity was positively correlated with perceived stress and was negatively correlated with diet quality. Study 1 tested whether greater perceived stress mediated the relationship between food insecurity and diet quality (Figure 2). The indirect effect of perceived stress was not significant. Although food insecurity was associated with higher perceived stress, higher perceived stress was not significantly associated with diet quality. Study 1 also examined whether perceived stress mediated the relationship between food insecurity and greater reward-based eating (Figure 3). The results showed that the indirect effect was significant, wherein food insecurity status was associated with higher perceived stress, and higher perceived stress was linked to higher reward-based eating. The squared standardized indirect effect of perceived stress indicated a small effect size (*υ* = 0.02) according to Cohen’s effect size benchmarks [44].

### 2.3. Study 1 Discussion

Study 1 revealed that greater perceived stress mediated the association between food insecurity and reward-based eating. The findings from this study align with animal models showing that food insecurity may alter reward processes and, to our knowledge, yield the first evidence of a correlational link between food insecurity and reward-based eating in humans. The results from Study 1 should be interpreted considering the characteristics of the analytic sample. The sample for Study 1 was composed exclusively of middle-aged Black and white women from Richmond, CA, which limits the generalizability of these findings to other demographic groups. It is possible that the observed associations may differ across men, individuals from other racial and ethnic backgrounds, and younger populations. These associations may vary in magnitude or direction across groups, as differences in structural conditions, resource usage, and coping resource availability may shape how food insecurity relates to eating behaviors. Future research could consider replicating the associations in diverse samples of individuals and assess how stress and coping with food insecurity may differ across people. Lastly, although the indirect effects were statistically significant, their magnitude was small per Cohen’s effect size benchmarks. These findings indicate that perceived stress explains only a portion of the association, leaving substantial variance to be explained by other unmeasured factors. Future research may consider identifying additional psychological and biological mediators that underlie the association between food insecurity and greater reward-based eating drive.

However, perceived stress did not mediate the association between food insecurity and diet quality. There are several possible explanations for the null associations. First, it is possible that subjective perceptions of stress are necessary but not sufficient to directly influence diet quality. Socioeconomic factors like household income and education level may have a stronger influence on diet-related outcomes. Indeed, income and education level were significant covariates in the models reported here. In a comparable study, food insecurity was not significantly associated with diet quality among a sample of African American and white adults in the Mississippi Delta Region [45]. Analogously to our study, income and education were significant predictors of diet quality [45]. Second, the discrepancy in recall periods for measures assessing food insecurity, perceived stress, and diet quality may have reduced our ability to detect any indirect effects of perceived stress, thus contributing to the null associations reported here. Food insecurity was assessed over the past 12 months, whereas diet quality only reflected the participant’s intake during a three-day period. As a result, the diet measure may not have adequately captured the cumulative impact of food insecurity on diet quality across the same period. Given the cyclical nature of food insecurity and food assistance programs [46], participants may have reported their diet quality in a period of relative abundance, when diet quality may not have been compromised. This temporal mismatch may have obscured the association between food insecurity and diet quality and any potential indirect effects of perceived stress. Future research should assess food insecurity, stress, and dietary outcomes over aligned time frames using longitudinal designs to provide a robust test of the proposed mediation pathway. These findings may also suggest that the psychological stress pathway linking food insecurity to poor outcomes is more relevant for certain eating behaviors than for overall dietary quality. Behaviors such as reward-based eating may be more directly responsive to perceptions of stress, whereas diet quality may be more strongly shaped by structural and economic constraints. This pattern suggests that the conceptual framework in which perceived stress operates as a mediator may be more relevant for eating behaviors than for overall diet-related outcomes, although further work is needed to confirm this distinction.

## 3. Study 2

Study 2 assessed whether perceived stress statistically mediated the association between food insecurity and comfort eating. Comfort eating is defined as eating or overeating hyperpalatable foods in response to negative emotion and can be used as a coping strategy to ameliorate negative psychological responses to adverse events and conditions [47,48]. Comfort eating is a distinct construct from reward-based eating in that it can occur in response to negative emotions, whereas reward-based eating can occur in their absence. A period of food insecurity, in theory, could be counted among those adverse events and conditions that trigger comfort eating [18]. Lopez-Cepero and colleagues reported that perceived stress explained almost 70% of the relationship between food insecurity and greater emotional eating (i.e., an inability to resist eating in response to negative emotions) in a sample of Latinx participants from Massachusetts [14]. Among a sample of women, perceived stress explained a significant amount of variance in the relationship between food insecurity and emotional overeating (i.e., eating in response to negative emotions) [13]. Thus, the current study examined whether perceived stress was a statistical mediator of the association between food insecurity and comfort eating.

### 3.1. Study 2 Materials and Methods

#### 3.1.1. Analytic Sample

Data were collected from respondents (*N* = 2022) recruited via a Qualtrics panel from December 2019 to February 2020 [49]. The sample was U.S. census-matched according to age, gender, income, race/ethnicity, and census region. Eligible respondents were at least 18 years of age and provided informed consent. Respondents were excluded if they incorrectly answered an attention check item; reported implausible values for height (i.e., <44 or >90 inches), weight (i.e., <55 or >1000 pounds), or body mass index (BMI) (i.e., BMI <12 or >70); failed Qualtrics data quality standards; or had a duplicate IP address. The final sample yielded a total of 1993 respondents (*M* = 47.22; *SD* = 19.96). Table 3 provides descriptive statistics of the analytic sample.

#### 3.1.2. Measures

Food Insecurity

Food insecurity was measured using the USDA’s U.S. Household Food Security Survey Module Six-Item Short Form [50]. Higher scores reflected a greater severity of food insecurity (0–6). Like Study 1, the food insecurity variable was dichotomized, wherein scores of zero reflected food security, and scores of one or greater reflected food insecurity.

Perceived Stress

Perceived stress was assessed using the 4-item version of the Perceived Stress Scale (α = .78) [4]. This brief version of the scale has been shown to be a validated measure when compared to the full, 10-item form [33].

Comfort Eating

Comfort eating was described to respondents as follows: “When feeling a negative emotion, some people eat more food and/or eat more unhealthy food than usual—a behavior described as comfort eating” [49]. The frequency of comfort eating was measured using the following question: “During the past 30 days, on how many days did you comfort eat?” Respondents were given the option to report 0 to 30 days of comfort eating. Scores ranged from 0 to 30 days of reported comfort eating.

#### 3.1.3. Covariates

As in Study 1, relevant socioeconomic and demographic confounds, including income, education, household size, race, gender, and age, were included as covariates. Race, gender, and education were dummy-coded.

#### 3.1.4. Statistical Analyses

Hypotheses and data analytic plans were pre-registered (OSF; https://osf.io/85hac/overview?view_only=c3e3a5d895fc43ffb55b728e9e93b5a2, accessed on 10 December 2020). Zero-order correlations were conducted between continuous variables to examine the relationship among variables of interest. Similarly to Study 1, missing data were handled using a Bayesian multiple imputation approach in rBlimp (0.2.25). Mediation models were estimated using a two-part count framework to account for the large number of zero values in the comfort eating outcome. Food insecurity status was included as a binary predictor and perceived stress included as a continuous mediator. Comfort eating was modeled using two components: (1) a binary component estimating the likelihood of reporting any comfort eating (i.e., any vs. none) and (2) a count component estimating the number of comfort eating days among participants who reported at least one day of comfort eating. Indirect effects were computed for the binary and count parts of the model. For the binary component, the indirect effect represents the extent to which food insecurity is associated with the likelihood of any comfort eating through perceived stress. For the count component, the indirect effect represents the extent to which food insecurity is associated with the frequency of comfort eating through perceived stress among those who engage in the behavior. Because the count model is nonlinear, conditional indirect effects were estimated separately for individuals with and without food insecurity. Indirect effects were computed as the product of the association between food insecurity and perceived stress (path a) and the association between perceived stress and each outcome component (path b). Like Study 1, the indirect effect calculated from these models represented the extent that the association between food insecurity and comfort eating is statistically accounted for by perceived stress. Models included age, gender, race, household size, income, and education as covariates, with race, gender, and education being dummy-coded. The models were estimated using two imputations, with 10,000 burn-in cycles followed by 20,000 posterior iterations across two Markov Chain Monte Carlo chains. A conditional indirect effect was considered statistically significant if the 95% credible interval did not include zero. Due to the two-part model including nonlinear components and conditional indirect effects, a standardized effect size comparable to those from linear mediation models was not computed. Similarly to Study 1, sensitivity analyses were conducted using the full food insecurity scale specified as an ordinal predictor. The results were substantively consistent, with no meaningful changes in statistical significance or interpretation. The results from models using the dichotomized food insecurity variable are reported.

### 3.2. Study 2 Results

**Figure 4:**
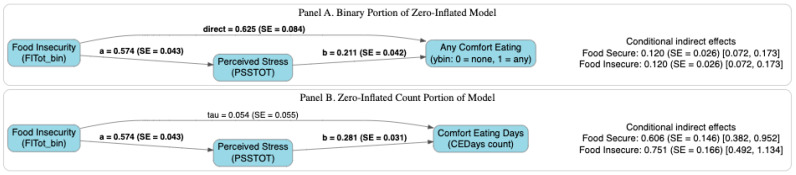
The two-part mediation model for the relationship between food insecurity and comfort eating. Note: Perceived stress statistically mediated the association between food insecurity and comfort eating. Higher food insecurity was associated with greater perceived stress, which in turn was associated with both a higher likelihood of any comfort eating (**A**) and greater frequency of comfort eating among those reporting at least one day (**B**). Panel A displays the binary portion of the model (occurrence of comfort eating; any vs. none), and Panel B displays the count portion (frequency of comfort eating among those reporting at least one day). The *a* paths represent the association between food insecurity and perceived stress. The *b* paths represent the association between perceived stress and the respective comfort eating outcome. For the count portion (**B**), the direct effect tau is reported on the log-count scale. All coefficients are unstandardized; binary model coefficients are on the probit scale, and count model coefficients are on the log-count scale. Conditional indirect effects are presented with 95% credible intervals. Bold indicates statistical significance (95% CI does not contain zero). *SE* = standard error; CI = credible interval.

Table 4 displays zero-order correlations for Study 2. Food insecurity was positively correlated with perceived stress and comfort eating. Study 2 leveraged a two-part mediation model to distinguish between the occurrence of comfort eating (any vs. none) and the frequency of comfort eating among individuals reporting at least one day (Figure 4). This model tested whether perceived stress mediated the association between food insecurity and the frequency of comfort eating. The results from the binary portion of the model indicated that food insecurity was associated with a greater likelihood of any comfort eating (versus not comfort eating) via higher perceived stress. Furthermore, among individuals who reported at least one day of comfort eating, higher perceived stress was significantly associated with a greater number of comfort eating days. This conditional indirect effect was observed among both food-secure and food-insecure individuals, indicating that perceived stress was associated with a greater frequency of comfort eating across groups. However, the indirect effect was slightly larger among individuals experiencing food insecurity, suggesting that the stress-related pathway linking food insecurity to comfort eating may be stronger in this group.

### 3.3. Study 2 Discussion

Study 2 leveraged data from a large census-matched U.S. sample to examine whether higher perceived stress mediated the association between food insecurity and the frequency of comfort eating. The results indicated that perceived stress partially explained the association between food insecurity and more frequent comfort eating, with a stronger effect observed among participants experiencing food insecurity compared with those who were food-secure. Research on the perceived benefits and frequency of comfort eating indicates that negative mood management, reduced boredom, and improved cognitive competence are associated with comfort eating frequency but not perceptions of comfort eating as pleasurable and accompanied by positive emotions [51]. Thus, individuals who perceive greater psychological stress may be more likely to manage their stress by engaging in comfort eating. Consistent with broader theoretical models of stress and coping, perceived stress is unlikely to influence comfort eating in isolation. Other psychological processes such as depressive symptoms, food-related anxiety, and emotion regulation difficulties may interact with perceived stress, thereby contributing to comfort eating. Notably, perceived stress is also associated with a higher frequency of comfort eating among food-secure individuals, suggesting that stress is a general psychological factor that is correlated with comfort eating. The results from Study 2 should be interpreted with certain limitations. Comfort eating was assessed using a single-item self-report measure, which may not fully capture the complexity of this behavior and may limit construct validity. The measure did not assess situational or emotional contexts that may influence comfort eating [52], potentially restricting the ability to capture variability in the behavior. As a result, this single-item measure may provide an incomplete representation of comfort eating. This limitation may introduce random measurement error and reduce the ability to adequately capture the construct of interest. Although the single-item measure was used to reduce participant burden, future work should consider using multi-item validated scales to more comprehensively assess comfort eating. Furthermore, effect sizes for the indirect effects estimated from the two-part zero-inflated mediation model were not calculated because the model contained nonlinear components (binary outcome and a zero-inflated outcome). Thus, the strength of the indirect effects should be interpreted in relative rather than standardized effects.

## 4. Strengths and Limitations

This investigation has several strengths. First, the studies provide novel evidence linking food insecurity to reward-based eating and comfort eating, two eating behaviors that have received limited empirical attention to date. By examining both behaviors, this study extends prior research that has largely focused on more general dietary patterns or disordered eating. Second, the findings identify perceived stress as a statistical mediator of the association between food insecurity and eating behaviors. These results offer preliminary support for the conceptual model being tested, wherein psychological factors like perceived stress, in addition to material and structural constraints, are correlated with food insecurity. Third, the analytic models accounted for important sociodemographic covariates including income, education, and household size. Adjusting for these factors strengthens confidence that the observed associations reflect processes that are not solely explained by the effects of socioeconomic status. Finally, Study 2 leveraged a census-matched U.S. sample, increasing the generalizability of the findings that identify perceived stress as a potential mediator of comfort eating in both food-secure and food-insecure individuals.

The results should be interpreted considering several conceptual limitations. First, data for both studies were cross-sectional, and thus, no causal or mechanistic claims can be made. The mediation models reported should therefore be interpreted as statistical associations among focal variables rather than evidence for causal pathways. Cross-sectional data may also obscure bidirectional relationships among the focal variables. For example, higher levels of perceived stress may contribute to reward-based and comfort eating, while these eating behaviors may, in turn, influence the levels of perceived stress. Furthermore, the cross-sectional nature of the data for both studies made it difficult to fully assess the temporal ordering between the focal variables. Future research can leverage longitudinal designs that assess the focal variables over comparable recall periods to better establish temporal ordering and assess potential bidirectional relationships. Additionally, the current conceptual model focused on perceived stress as the sole psychological mediator. The current work did not assess relevant constructs like mental health symptoms or coping strategies. Meta-analytic evidence indicates that food insecurity is correlated with depression and anxiety [53], both of which may also contribute to reward-based eating and comfort eating behaviors [54,55]. Theoretical models of stress and coping further suggest that individuals may engage in cognitive and behavioral coping responses, such as emotion regulation or engagement in social support resources, in response to stress-related experiences [56]. Future research could benefit from incorporating multiple statistical mediating or moderating factors alongside perceived stress to provide a comprehensive account of these associations.

Additional methodological limitations should be considered. First, all focal variables were assessed via self-report questionnaires. This approach is subject to recall error and social desirability bias, particularly for sensitive constructs such as food insecurity and dietary behaviors. Future studies could use ecological momentary assessment to capture food insecurity, perceived stress, dietary intake, and eating behaviors repeatedly across the day, thereby reducing retrospective bias and improving ecological validity. Second, data for Study 2 were collected using an online survey. Web surveys are susceptible to self-selection bias as well as the under-coverage of underrepresented individuals. Future research should replicate these findings using probability-based samples or mixed-methods data collection approaches to better capture individuals who may be underrepresented in web-based surveys. Third, the two studies used different mediation analytic approaches, which limits the comparability of indirect effects across studies. The results should be interpreted as preliminary support for a broader conceptual framework in which perceived stress operates as a statistical mediator, rather than as evidence of differences in effect magnitude across eating-related outcomes. Additionally, food insecurity was dichotomized in both studies. This approach improved interpretability but may have reduced variability in food insecurity severity. Consequently, potential dose–response associations between food insecurity and the outcomes may have been obscured. Future research may consider refining the current conceptual model to assess how food insecurity severity may alter relationships to perceived stress, diet, and eating behaviors.

## 5. Conclusions

Food insecurity is associated with a range of adverse health outcomes that may arise not only from limited economic resources but also from the psychological strain of living in such conditions. The present findings identify perceived stress as a common correlate of both food insecurity and maladaptive eating behaviors and provide preliminary, associative evidence linking food insecurity to eating behaviors through perceived stress. Given the cross-sectional nature of the data and differences across studies, these findings should be interpreted cautiously. These findings may have practical implications for public health and intervention design. Incorporating stress screening or stress management components into food assistance programs may represent a feasible and scalable approach to addressing both the material and psychosocial aspects of food insecurity. Future research using longitudinal and experimental designs is needed to clarify causal relationships and to determine whether targeting perceived stress may be an effective component of interventions addressing food insecurity.

## Figures and Tables

**Table 1:** Sociodemographic characteristics of participants in Study 1.

Sociodemographic Variable	Food-Secure (*n* = 303)	Food-Insecure (*n* = 71)
Age	39.3 (1.2)	39.6 (1.1)
Household Size	3.6 (1.6)	3.6 (1.8)
Race		
Black	146 (48.2)	39 (54.9)
White	157 (51.8)	32 (45.1)
Household Income		
Less than $50,000	86 (29.8)	41 (60.4)
$50,000–$99,999	96 (33.1)	24 (35.2)
$100,000–$149,999	58 (20.1)	0
Greater than $150,000	49 (17)	3 (4.4)
Highest Education Level		
High school or less	59 (19.5)	20 (28.2)
Some college	107 (35.3)	39 (54.9)
College or more	137 (45.2)	12 (16.9)

Note. This table is based on non-imputed data. Values are presented as mean (SD) for continuous variables and *n* (%) for categorical variables. Missing data reported for household size (*n* = 9), household income (*n* = 40), and education level (*n* = 1). Household income is presented as a categorical variable for descriptive statistics but was analyzed as a continuous variable in mediation analyses.

**Table 2:** Zero-order correlations between variables of interest for Study 1.

Measure	1	2	3	4	5	6	7
1. Age	--						
2. Food Insecurity	0.06	--					
3. Reward-Based Eating	**0.08**	0.07	--				
4. Perceived Stress	−0.06	**0.30**	**0.21**	--			
5. Diet Quality Score	0.00	**−0.11**	**0.11**	**−0.14**	--		
6. Household Size	0.00	0.02	0.00	0.07	−0.07	--	
7. Income	−0.01	**−0.33**	**0.10**	**−0.22**	**0.27**	0.05	--
Mean	39.52	1.20	0.77	17.56	56.70	3.63	7.40
*SD*	1.28	0.40	0.77	6.85	14.18	1.66	4.89
Min–Max	36–43	1–2	0–4	0–39	21.78–101.8	1–14	1–15

Note. Values represent Pearson correlation coefficients. Means, standard deviations (*SD*), and ranges are reported for each variable of interest. Analyses are based on non-imputed data. Bold indicates statistical significance (*p* < .05).

**Table 3:** Sociodemographic characteristics of participants in Study 2.

Demographic Variable	Food-Secure (*n* = 947)	Food-Insecure (*n* = 722)
Age	54.4 (16)	40.7 (15.3)
Gender		
Woman	472 (49.8)	369 (51.1)
Man	472 (49.8)	351 (48.6)
Non-binary/Other	3 (0.3)	2 (0.3)
Race/Ethnicity		
White	744 (78.6)	356 (49.3)
Black or African American	61 (6.4)	139 (19.3)
Native American, Eskimo, or Aleut	6 (0.6)	16 (2.2)
Hispanic or Latino/a	79 (8.3)	154 (21.3)
Asian or Asian American	49 (5.2)	38 (5.3)
Native Hawaiian or Pacific Islander	0 (0)	2 (0.3)
Biracial or multiracial	6 (0.6)	13 (1.8)
Other	2 (0.2)	4 (0.6)
Household Income		
Less than $50,000	247 (26.1)	387 (53.6)
$50,000–$99,999	373 (39.4)	209 (28.9)
$100,000–$149,999	173 (18.3)	88 (12.2)
Greater than $150,000	154 (16.2)	38 (5.3)
Highest Education Level		
High school or less	140 (14.8)	213 (29.5)
Some college but no degree	237 (25)	177 (24.5)
Associate degree	122 (12.9)	111 (15.4)
Bachelor’s degree	274 (28.9)	138 (19.1)
Graduate or doctoral degree	174 (18.4)	83 (11.5)

Note. Table is based on non-imputed data. Values are presented as mean (SD) for continuous variables and *n* (%) for categorical variables. Missing data for education level (*n* = 227). Household income is presented as a categorical variable for descriptive statistics but was analyzed as a continuous variable in mediation analyses.

**Table 4:** Zero-order correlations between variables of interest in Study 2.

Measure	1	2	3	4	5	6
1. Age	--					
2. Food Insecurity	**−0.39**	--				
3. Perceived Stress	**−0.32**	**0.42**	--			
4. Comfort Eating	**−0.11**	**0.19**	**0.29**	--		
5. Household Size	**−0.32**	**0.12**	**0.14**	**0.10**	**--**	
6. Income	**0.04**	**−0.28**	**−0.16**	0.01	**0.16**	**--**
Mean	47.22	0.45	2.66	6.55	2.68	3.24
*(SD)*	17.30	0.50	0.83	8.14	1.49	1.74
Min–Max	18–87	0–1	1–5	0–30	1–25	1–7

Note. Values represent Pearson correlation coefficients. Means, standard deviations (*SD*), and ranges are reported for each variable of interest. Analyses are based on non-imputed data. Bold indicates statistical significance (*p* < .05).

## Data Availability

Data available on request due to restrictions (e.g., privacy, legal or ethical reasons). Study 1 data derive from the National Heart, Lung, and Blood Institute Growth and Health Study. Historical data are publicly available through the NHLBI BioLINCC repository (https://biolincc.nhlbi.nih.gov/). The specific data used in this study are not publicly posted but may be available as de-identified data upon reasonable request, subject to institutional review board approval and a formal data use agreement. Requests should be directed to B.A.L (blaraia@berkeley.edu) or E.S.E (elissa.epel@ucsf.edu). A de-identified analytic dataset and R code for Study 2 sufficient to reproduce the reported analyses will be deposited in an open repository (i.e., OSF) upon completion of repository access transition. In the interim, materials are available from the corresponding author upon reasonable request.
